# Children and youth perceive smoking messages in an unbranded advertisement from a NIKE marketing campaign: a cluster randomised controlled trial

**DOI:** 10.1186/1471-2431-11-26

**Published:** 2011-04-08

**Authors:** Nathalie Auger, Mark Daniel, Bärbel Knäuper, Marie-France Raynault, Barry Pless

**Affiliations:** 1 Institut national de santé publique du Québec, 190, boulevard Crémazie Est, Montréal, Québec H2P 1E2, Canada; 2 Research Centre of the University of Montréal Hospital Centre, 3850, rue Saint-Urbain, Montréal, Québec H2W 1T7, Canada; 3 Department of Social and Preventive Medicine, University of Montréal, PO Box 6128, succursale Centre-ville, Montréal, Québec H3C 3J7, Canada; 4 School of Health Sciences, University of South Australia, GPO Box 2471, Adelaide, South Australia 5001, Australia; 5 Department of Psychology, McGill University, 1205 Dr. Penfield Avenue, Montréal, Québec H3A 1B1, Canada; 6 Montréal Public Health Department, Régie régionale de Montréal-Centre, 1301, rue Sherbrooke Est, Montréal, Québec H2L 1M3, Canada; 7 Departments of Pediatrics, Epidemiology and Biostatistics, McGill University, 2155 Guy Street, 5th Floor, Montréal, Québec H3H 2R9, Canada

## Abstract

**Background:**

How youth perceive marketing messages in sports is poorly understood. We evaluated whether youth perceive that the imagery of a specific sports marketing advertisement contained smoking-related messages.

**Methods:**

Twenty grade 7 to 11 classes (397 students) from two high schools in Montréal, Canada were recruited to participate in a cluster randomised single-blind controlled trial. Classes were randomly allocated to either a NIKE advertisement containing the phrase 'LIGHT IT UP' (n = 205) or to a neutral advertisement with smoking imagery reduced and the phrase replaced by 'GO FOR IT' (n = 192). The NIKE logo was removed from both advertisements. Students responded in class to a questionnaire asking open-ended questions about their perception of the messages in the ad. Reports relating to the appearance and text of the ad, and the product being promoted were evaluated.

**Results:**

Relative to the neutral ad, more students reported that the phrase 'LIGHT IT UP' was smoking-related (37.6% vs. 0.5%) and that other parts of the ad resembled smoking-related products (50.7% vs. 10.4%). The relative risk of students reporting that the NIKE ad promoted cigarettes was 4.41 (95% confidence interval: 2.64-7.36; P < 0.001).

**Conclusions:**

The unbranded imagery of an advertisement in a specific campaign aimed at promoting NIKE hockey products appears to have contained smoking-related messages. This particular marketing campaign may have promoted smoking. This suggests that the regulation of marketing to youth may need to be more tightly controlled.

## Background

Large corporations use increasingly sophisticated marketing strategies to promote products to children, which includes marketing techniques that rely on imagery relating to lifestyle or social norms. Such forms of marketing are acknowledged more and more as important determinants of child health that need to be regulated. Several countries have implemented mechanisms to regulate marketing to children, especially with respect to the promotion of tobacco [1]. One of these countries is Canada, which has led the way in regulating tobacco marketing [2], particularly because the tobacco industry has used such marketing techniques so effectively with children [3-7]. Evidence also shows that targeting of children by the food industry may be fuelling the obesity epidemic [8,9]. Scant research has, however, considered whether marketing by other industries may influence the health of children. Large corporations that market heavily may be popular with children and can shape their thoughts and behaviours, possibly even when marketing laws are present. There is a need to evaluate how children perceive marketing campaigns as a first step towards understanding how to improve marketing policies in general.

The goal of the current study was to assess what youth perceive in the imagery of advertisements used in a specific marketing campaign by NIKE, a company that is popular with children and youth. We evaluated NIKE's LIGHT IT UP campaign, run from 2003-2005 in Canada to "inspire young hockey players" [10]. This particular campaign was selected because it was meant to promote hockey products yet appeared to include messages that could have inadvertently promoted smoking. This is of concern because it has been shown that the tobacco industry uses sports to promote its products [6,11-13].

## Methods

### Study design and context

We used a randomised single-blind controlled trial design. Twenty grade 7 to 11 classrooms from two schools were allocated to receive either the exposure advertisement (10 classes, 205 students) or a neutral version of the ad (10 classes, 192 students). We consulted youth tobacco control experts who recommended an assessment without the swoosh logo to determine how youth perceived the internal imagery of the ads independent of the brand name. Hence, the Nike logo was removed from both the exposure and control advertisements. Approval for this study was obtained from the research ethics committee of the University of Montréal Hospital Centre.

### Participants

We aimed for a sample of 538 students (269 for each condition) assuming a design effect of 1.11, with 5% of the exposed students and none of the control students perceiving tobacco-related messages (77% participation rate, two-sided α = 0.05, β = 0.10, intraclass correlation = 0.005 for students within classrooms). In February 2009, 522 students from one junior and one senior high school in the metropolitan area of Montréal, Canada were invited to participate. Voluntary signed consent was obtained for 401 students and from their parents three weeks prior to the test date. Three students were absent on the test date and one did not follow the protocol, leaving 397 participants (71.9% participation rate).

### Procedures

The original campaign was Internet-based. Messages were promoted on NIKE's homepage in a web-based multimedia presentation providing links to photos and videos of children posing next to LIGHT IT UP ads, and to screensavers/wallpapers. Children were recruited to the web-site by NIKE representatives in arenas, tournaments, skating rinks, hockey practices and retail locations in Toronto, Montreal, Calgary and Vancouver. Ads contained a hockey net referred to as the "lonely talking net", and were available for download for non-commercial purposes.

**Figure 1:**
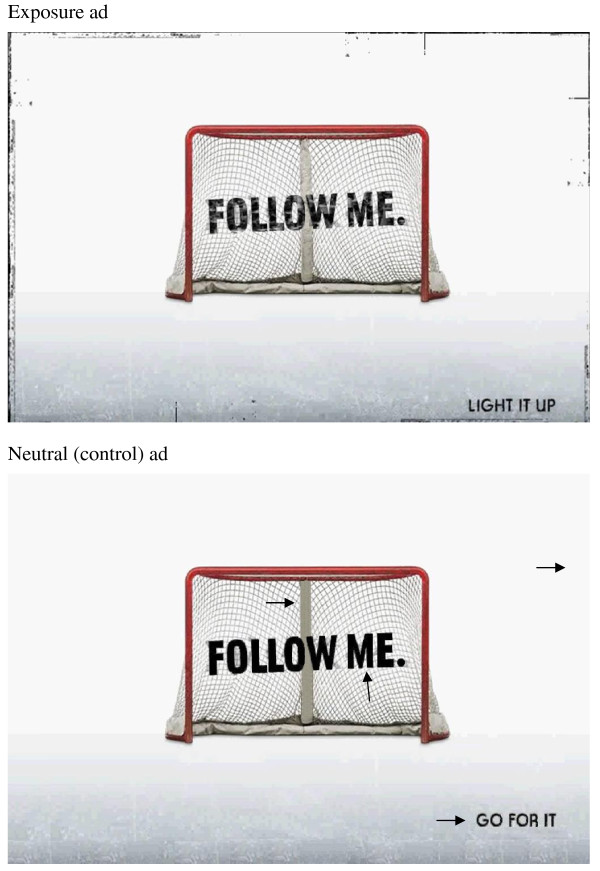
**Images of the ads shown to students**. Figure 1A. Exposure ad. Figure 1B. Neutral (control) ad. Arrows point to digitally modified areas (1 - central pole was coloured grey using a shade from the lower part of the pole; 2 - FOLLOW ME was blackened; 3 - rectangular marks on outmost edges were removed; 4 - LIGHT IT UP was replaced by GO FOR IT). ^© ^of the original image: NIKE, Inc.

We used an image containing the message FOLLOW ME and the slogan LIGHT IT UP as the exposure, without the logo (Figure 1a, ^© ^of the original image: NIKE, Inc). This image was taken from the multimedia presentation and resembled most of the other ads on the web-site, including those meant to be downloaded and used as computer background wallpaper by youth. In consultation with colleagues, we identified four parts of the image potentially containing tobacco-related imagery, including the 1) slogan, 2) ash-like appearance of the net center pole, 3) smoky appearance of the words in the center, and 4) unusual rectangular marks around the border that resembled cigarettes. We generated a neutral comparison ad as the control using Windows Paint software. In the control, we reduced possible tobacco-related content as follows: 1) the LIGHT IT UP slogan was changed to GO FOR IT; 2) the colour of the net center pole was changed to a uniform grey taken from the bottom of the net; 3) the FOLLOW ME was blackened; and 4) the rectangular marks in the outermost edges of the ad were removed (Figure 1b). For simplicity, the unbranded NIKE image is hereafter referred to as the exposure advertisement, and the control as the neutral advertisement.

We designed a 3-part questionnaire to determine the types of messages perceived. Part 1 contained open-ended questions asking for impressions of the ad, thoughts on the slogan, thoughts on the ad's appearance, the product or service being promoted, and the type of company they thought had produced the ad (Additional file 1). No mention of tobacco was made in any of these questions. Parts 2 and 3 contained multiple-choice questions about tobacco and baseline covariates potentially related to perception (age, sex, grade, socio-economic status, interest in hockey, and smoking). Seventeen grade 7 to 11 youth tested the questionnaire for question comprehension [14]. We assessed socio-economic status with the Family Affluence Scale [15], and smoking status using Pierce's method (non-susceptible never smoker, susceptible never smoker, experimenter, established smoker) [5]. Questionnaires were administered in classrooms under the supervision of a teacher and a research assistant. Students responded to each part in sequence without discussion with peers, sealing each part in separate envelopes before moving on to the next.

The research assistant randomly assigned classes to either the exposure ad or neutral ad within grade levels. Students were told different ads were being tested but were blinded as to which version they had been assigned and to our interest in the smoking question. As well, none of the teachers or school administrators was aware of the tobacco-related hypothesis.

### Outcome measures

Three primary outcomes, expressed as dichotomous variables, were defined as any report of pro-tobacco or sports-related messages in the 1) slogan, 2) ad's appearance, or 3) product being promoted. Reports were considered smoking-related when they included the specific terms "cigarette", "smoking", or "smoke". Reports were considered sports-related if they made any direct or remotely indirect reference to physical activity, physical fitness, health, skating, hockey or hockey product, or any other sport or game.

A trained research assistant extracted all tobacco- or sports-related messages from the first part of the questionnaire. Because the research assistant could not be blinded because of the slogan used in the questionnaire, a second assistant re-coded a 10% sample of questionnaires to determine whether similar results would be obtained. Percentage agreement between coders ranged from 96% to 100% for each outcome.

### Statistical analysis

Generalised estimating equations with a logit link were used to evaluate the association between the ad and reports of tobacco- or sports-related messages, accounting for classroom clustering. Finner P-values were computed with WinPEPI software to adjust for the multiple outcomes tested [16]. Relative risks (RR) with 95% confidence intervals (CI) were estimated. Multivariate models adjusting for sex, grade, socio-economic status, smoking status, test time and a test time-ad version interaction term were also run. Analyses were performed using SAS 9.1 software (SAS Institute Inc., Cary, NC, 2002).

## Results

As shown in Table 1, the characteristics of students in both groups were highly similar although 11% more students shown the exposure ad were tested in the morning. The mean age of both groups was 14 years, with females, non-smokers, and ice hockey fans more frequently represented.

**Table 1:** Baseline characteristics of students

Characteristic	Exposure ad (N = 205)	Neutral ad (N = 192)	P-value
**Age, mean (SD)**	14.0 (1.4)	14.1 (1.7)	0.86*
**Male, n (%)**	83 (40.5)	76 (39.6)	0.85
**Grade, n (%)**			
7	54 (26.3)	56 (29.2)	0.43
8	47 (22.9)	44 (22.9)	
9	60 (29.3)	50 (26.0)	
10	23 (11.2)	14 (7.3)	
11	21 (10.2)	28 (14.6)	
**Family affluence score, mean (SD)**^†^	5.5 (1.2)	5.4 (1.2)	0.19*
**Smoking status, n (%)**			
Nonsusceptible never smoker	119 (58.6)	96 (50.5)	0.30
Susceptible never smoker	30 (14.8)	40 (21.1)	
Experimenter	43 (21.2)	41 (21.6)	
Established smoker	11 (5.4)	13 (6.8)	
**Ice hockey fan, n (%)**			
Very much	67 (32.8)	64 (33.5)	0.30
A bit	55 (27.0)	60 (31.4)	
Not really	47 (23.0)	30 (15.7)	
No	35 (17.2)	37 (19.4)	
**Test time, n (%)**^‡^			
Morning	166 (81.0)	134 (69.8)	0.01
Afternoon	39 (19.0)	58 (30.2)	

* P-values are based on the Wilcoxon rank sum test for two variables; all other p-values are based on contingency tables for categorical variables.

† Ranges from 1 to 7

‡ Grade 7 and 8 questionnaires were administered during one class period. Grade 9 to 11 questionnaires were administered either in the morning or afternoon.

### Tobacco versus sports content of ads

One third (37.6%, 77/205) of students viewing the LIGHT IT UP version thought the slogan referred to smoking compared with 0.5% (1/192) who viewed the GO FOR IT version (Table 2). Many more students also reported that the exposure ad relative to the neutral ad contained images of smoking-related products (50.7%, 104/205 vs. 10.4%, 20/192; RR 4.87, 95% CI 2.86-8.29). Examples of smoking-related reports were that that the centre pole resembled a cigarette, that smoke covered the central text of the ad, and that cigarettes were present in the ad's edge. Students also reported that the product being promoted by the exposure ad relative to the neutral ad was cigarettes (39.0%, 80/205 vs. 8.9%, 17/192; RR 4.41, 95% CI 2.64-7.36).

**Table 2:** Relative risks for reporting tobacco messages for NIKE ad versus neutral ad*

Outcome	Exposure ad (N = 205) n (%)	Neutral ad (N = 192) n (%)	Relative risk (95% confidence interval)	P-value
**Presence of smoking-related messages**				
Slogan refers to smoking	77 (37.6)	1 (0.5)	72.1 (10.3-503.5)	<0.001
Ad contains images of smoking-related products	104 (50.7)	20 (10.4)	4.87 (2.86-8.29)	<0.001
Ad is promoting cigarettes	80 (39.0)	17 (8.9)	4.41 (2.64-7.36)	<0.001

* Data represent self-reported tobacco messages from open-ended questions

A lower than expected number of students reported the ads were sports-related (Table 3). For both ads, only one-third of students reported the slogan referred to sports and only half reported the ad had a sports-related appearance. However, students were more likely to report that the neutral ad promoted sports relative to the exposure ad (65.8%, 125/192 vs. 51.0%, 103/205; RR 0.78, 95% CI 0.68-0.89).

**Table 3:** Relative risks for reporting sports-related messages for NIKE ad versus neutral ad*

Outcome	Exposure ad (N = 205) n (%)	Neutral ad (N = 192) n (%)	Relative risk (95% confidence interval)	P-value
**Presence of sports-related messages**				
Slogan refers to sports	78 (38.1)	67 (34.9)	1.09 (0.80-1.49)	0.56
Ad contains images of sport-related products	52 (25.4)	50 (26.0)	0.97 (0.72-1.31)	0.90
Ad is promoting sports	103 (51.0)	125 (65.8)	0.78 (0.68-0.89)	0.0045

* Data represent self-reported sports messages from open-ended questions

Accounting for age, sex, grade, socio-economic status, smoking status, being a hockey fan, and test time did not influence the relationship between ad version and perception of tobacco or sports messages. Inclusion of a test time*ad version interaction term in models did not change the results, nor did models run after excluding afternoon students. Hence, there is no evidence that senior students participating during afternoon test classes were biased by morning class students to whom they might have spoken (this issue did not apply to students from the junior high school who were all tested during one class period). Only one participant (0.5%) reported having seen similar ads in the past, and none reported this was a NIKE ad.

Written comments of students shown the exposure ad are provided in Table 4. Students from all grades reported that the exposure ad made them think "about cigarettes", that LIGHT IT UP meant "light up your cigarette", that the centre pole "really looks like a cigarette", that the ad was "obviously" promoting cigarettes and was about "smoking, disguised as a hockey ad", and even that an "illegal company or a cigarette company" had made the ad. A minority of students who reported tobacco messages for the neutral ad mainly said the centre pole resembled a cigarette (comments not shown).

**Table 4:** Examples of statements on what students thought of the exposure NIKE ad*

Quote	Grade
**First impression of image**	
"It makes me think of a cigarette commercial that is trying to influence young teens/adults to smoke."	7
"I saw the cigarette as the pole and I knew it meant smoking."	7
"It's about smoking and that smoking should be your goal."	8
"This ad first reminds me of cigarettes and smoking."	10
**Meaning of LIGHT IT UP**	
"I've heard that expression for lighting a cigarette."	7
"They're trying to use hockey as an image of fun, then they use a cigarette in the hockey net, then they add LIGHT IT UP. Therefore, they want you to start smoking."	8
"To light up your cigarette."	9
"It can either mean to light up a cigarette or drug and then you'll become successful or it can mean give the game all you got."	10
"I think about opening a lighter."	11
**Meaning of FOLLOW ME**	
"To smoke because your friends are doing it and if they offer you, say yes."	8
"FOLLOW ME would be 'drawing in' teens to smoke. The sign to me encourages young teens to start and develop a smoking habit, portraying it as a good thing."	10
"Follow the person smoking."	11
**Appearance of FOLLOW ME**	
"There is smoke within the black lettering."	7
"It looks like smoke."	9
**Appearance of Centre pole of hockey net**	
"It looks like a cigarette only without the orange thing at the butt of the cigarette."	8
"The centre pole really looks like a cigarette or drugs."	11
**Appearance of Outermost edges of image**	
"I see little cigarettes that look like they're already lit up."	7
"I can see cigarettes on all of the sides in a faded looking way." (*Student also drew a picture)	9
"Cigarette filters."	11
**Product being promoted**	
"It is promoting for sure 'cigarettes'."	7
"People are advertising smoking."	8
"Obviously cigarettes."	9
"Smoking, buying cigarettes, getting addicted so the company can continue making money."	9
"Promoting the use of cigarettes or other things you can smoke."	10
"Cigarettes through sports. A lot of people watch hockey and even though we're not aware of it our brain picks up on the message it's sending."	11
"Easy, it's smoking, disguised as a hockey ad. You know, get all the cool athletes to smoke so it looks cool to the younger kids."	11
**Type of company that made the ad**	
"Export (I think that's a cigarette brand)."	7
"A cigarette company, a drug company."	8
"An illegal company or a cigarette company."	9
"Any cigarette company, people that benefit from cigarette production."	10
"Du Maurier, Peter Jackson, cigarette companies."	11

* Quotes are mutually exclusive (i.e., no student appears more than once in the list).

## Discussion

This randomized trial shows clearly that an ad image used by NIKE to associate its products with scoring in hockey was thought to promote smoking by one third of adolescents who saw it without the brand name. Though these results pertain to only one campaign, they nonetheless illustrate the potential for messages such as LIGHT IT UP to unintentionally promote tobacco to young people. This finding is important not only because tobacco is a leading cause of morbidity and mortality worldwide, but because smoking habits are formed during childhood and tobacco promotion is partly responsible [17].

Over a third of students (38%) reported the slogan LIGHT IT UP was related to smoking. We expected the youth in this study would associate these ads mainly with sports, as the ads were promoted by a well-known sports company that is expected to have carefully developed and tested their ads before going to market. Perception of sports-related messages was, however, no more frequent than perception of tobacco-related messages. A nearly similar ad that we designed to be equally vague but more tobacco neutral was significantly less likely to lead to reports of tobacco messages. Although the phrase LIGHT IT UP may have been intended to refer to lighting the scoreboard with a goal or to associate NIKE products with winning, even this is unclear: associations did not change with adjustment for being a hockey fan (hockey fans would be expected to understand the hidden meaning of LIGHT IT UP). Furthermore, the French version of the phrase (BRULE LA GLACE, or burn the ice), in no way alludes to scoring. Such messages may therefore be ambiguous and from a young person's viewpoint the interpretation may not be benign.

An important issue is that we removed the NIKE swoosh mark logo from the ads to determine whether students could correctly identify the category of product being promoted (i.e., sports) as it was not clear they would perceive tobacco messages in the first place, and to isolate the effects of the slogan and pictorial aspects of the ads. It is possible that fewer students might have reported tobacco-related messages with the NIKE brand logo kept in the ads, and future research using the same ads with the swoosh mark retained would be informative. Research on cigarette ads suggests that youth focus on the product being promoted rather than on the brand name, and that brand names may contribute little to the understanding of what product is being promoted [18]. Thus, the removal of the NIKE check mark in our study is not likely to fully account for so many students seeing tobacco in the ad. Furthermore, our procedure did not entirely differ from some of NIKE's own marketing behaviour, as the NIKE check mark was not visible in some of the ads shown to youth. It is not certain that inclusion of the logo in this study would have correctly represented the spectrum of ads shown to youth.

Without the logo, both the exposure ad and the neutral ad should have at least induced sports-related thoughts, given that an important goal of advertisements is to lead consumers to the correct product category. Students, however, reported that the neutral ad promoted sports more so than did the exposure ad. The small proportion of students who reported that the centre pole of the neutral ad resembled a cigarette is not unexpected because the grey shade is from the original ad.

Interestingly, a randomised study resembling ours showed that the text and colours of ads can change the perception of tobacco messages, but this research was done with adults and the ads were intentionally related to tobacco [19]. Our study shows that such factors may be important in ads targeting youth, and even important in ads not intentionally promoting tobacco. Some researchers have critiqued studies that evaluate the influence of the media by claiming that youth are less cognitively complex than adults, and hence less likely to pay attention to media messages [20]. In fact, the size of our sample was calculated assuming that few students would perceive tobacco imagery. Thus, our findings not only show that we should not underestimate the cognitive abilities of youth, but they also call into question theories that minimize the influence of advertisements by arguing that youth pay little attention to the media around them [20].

A related issue is how younger children would have perceived these ads. Although NIKE stated they surveyed 15 to 25 year olds before the campaign [10], according to images on the web-site elementary school aged children in particular were targeted. This study was designed for secondary school students, and further research is necessary to determine whether younger children would also perceive tobacco-related messages.

Our conclusions are limited by our inability to evaluate elements of the marketing campaign that were hidden to us. We could not enter the password-protected parts of the web-site that were only accessible to children who had registered at a sponsored promotional event, or who belonged to a hockey team sponsored by NIKE. We were not successful in attending such events and wrote to NIKE requesting a password to access the restricted sites but were refused. Thus, we could not determine the full spectrum of ads that were shown to children. The ads that we did find alluded to sexuality ("Guys think about scoring every six seconds", "Slip it between the legs"), risk-taking ("Some lines are meant to be crossed"), peer acceptance ("You are not alone", "Do you want in?"), and independence ("Are you ready to break free?"). These themes have successfully been used to market cigarettes to youth [6,11,21], with the tobacco industry finding innovative ways of encouraging repeated viewing of such ads (e.g., through contests) [6,22]. In the LIGHT IT UP campaign, NIKE ran a contest in which youth were required to repeatedly watch photos and videos of children posing next to the LIGHT IT UP messages [10]. We did not evaluate how these added factors could have influenced the perception of tobacco-related messages.

It is now established that tobacco advertising leads to smoking in youth [3-5]. Incidental pro-tobacco imagery on the Internet, in film and magazines is also increasingly linked to youth smoking [23-26]. Such messages shape social values about smoking and create environments where cigarettes are considered normal [24,25], and sports marketing campaigns not related to tobacco can potentially contribute to this process especially when the sport in question is popular [27]. NIKE also relied on hockey sponsorship to recruit children to the LIGHT IT UP campaign, and we do not know how their sponsorship strategies could have contributed. It is interesting to note that tobacco sponsorship per se was banned in 2003 by the Canadian Tobacco Act [2], just before the LIGHT IT UP campaign was run.

These issues are important because the tobacco industry has demonstrated that the combination of sponsorship, sports and tobacco works to promote cigarette smoking [6,11-13,28], and celebrities or athletes [10] may be enhancing factors [25,29]. NIKE also donated hockey equipment from the LIGHT IT UP campaign to disadvantaged children, and disadvantaged children are already at greater risk of smoking [30]. More research is needed on campaigns such as LIGHT IT UP to determine what kind of influence the factors outlined above could have on inadvertent tobacco promotion in different settings.

## Conclusions

We found that children and youth perceived smoking messages in a randomised trial testing unbranded ad imagery used in a NIKE marketing campaign run in four large Canadian metropolitan centers. Though these findings cannot be generalized to other marketing campaigns by NIKE or other sports companies, they nonetheless suggest that elements of a NIKE advertisement may have inadvertently promoted smoking among at least a small portion of the youths who were exposed, and that Canadian regulations for marketing to children and youth [2,31] may be inadequate when marketing relies on imagery with double meanings. Increasingly complex and hard-to-regulate marketing environments are emerging [8,9,32], and marketing regulations must keep pace with changing environments [33]. In particular, regulations for marketing on the Internet must be tightened. Large corporations must be accountable for their actions, including any inadvertent harmful effects of promotional efforts. Marketing must be transparent and easily accessible to adults. Until marketing regulations are improved and properly enforced, the public health and practising pediatric community needs to be vigilant regarding all marketing to children and youth.

## Competing interests

The authors declare that they have no competing interests.

## Authors' contributions

NA and MD conceived and designed the study, with assistance from BK and BP. MFR contributed to initial study conception. NA collected and analysed the data, and wrote the manuscript. MD, BK, MFR and BP revised the manuscript for important intellectual content. All authors read and approved the final manuscript. NA is the guarantor for the study.

## Pre-publication history

The pre-publication history for this paper can be accessed here:

http://www.biomedcentral.com/1471-2431/11/26/prepub

## Supplementary Material

Additional file 1 **Open-ended questions asked to students in Part 1 of the questionnaire**. List of open-ended questions asked to students in Part 1 of the questionnaire Click here for file
