# Supplementary material for: Children and youth perceive smoking messages in an unbranded advertisement from a NIKE marketing campaign: a cluster randomised controlled trial
Source: BMC Pediatr. 2011 Apr 8;11:26. doi: 10.1186/1471-2431-11-26 (PMC3087678; doi:10.1186/1471-2431-11-26)
Supplement: Additional file 1 — Open-ended questions asked to students in Part 1 of the questionnaire. List of open-ended questions asked to students in Part 1 of the questionnaire [file 1471-2431-11-26-S1.DOC]

**Additional files**

**Additional file 1 – List of open-ended questions asked to students in Part 1 of the questionnaire**

| 1. What is your first impression of this ad? |
| --- |
| 2. What are some things this ad makes you think of? |
| 3. Look at the ‘LIGHT IT UP / GO FOR IT’ on the bottom right of the ad. How do you interpret ‘LIGHT IT UP / GO FOR IT’? |
| 4. Can you think of other meanings for the ‘LIGHT IT UP / GO FOR IT’ in the ad? |
| 5. Look at the ‘FOLLOW ME’. What are some interpretations of the ‘FOLLOW ME’? You can use examples to explain. |
| 6. Now have another look at the ‘FOLLOW ME’. Is there anything interesting about the look of the ‘FOLLOW ME’? |
| 7. Look at the centre pole. What does the centre pole look like? |
| 8. Look at the outermost sides of the ad (not the sides of the net). Can you see the outline of any objects in the four sides? If so, what do you see? |
| 9. What products and/or services do you think the ad might be promoting? |
| 10. What kind of company do you think made this ad? You can write more than one. |
| 11. Does this ad make you think of anything else? |
